# Supplementary material for: Diversity and Antimicrobial Resistance in the Streptococcus bovis/Streptococcus equinus Complex (SBSEC) Isolated from Korean Domestic Ruminants
Source: Microorganisms. 2021 Jan 4;9(1):98. doi: 10.3390/microorganisms9010098 (PMC7824528; doi:10.3390/microorganisms9010098)
Supplement: Supplementary file 1 [file microorganisms-09-00098-s001.pdf]

Supplementary Materials

Table S1. Chemical composition (g/kg DM or as stated) of the ruminant diet fed to two fistulated Holstein cow.

Item†	Corn silage	Concentrate mix
DM (g/kg as fed)	921	878
OM	922	941
CP	121	171
EE	25	35
Ash	78	59
NFE	472	645
CF	304	90
Total carbohydrates	776	735
Ca	0.04	0.11
P	0.04	0.05

†DM, dry matter; OM, organic matter; CP, crude protein; EE, ether extract; NFE, nitrogen-free extract; CF, crude fiber.

Table S2. Primers used in this study

Gene	Primer sequence (5′ to 3′)	Annealing temperature (°C)	Amplicon size (bp)	Reference
Bacterial identification				
16S rRNA (for amplification)	F(27F): AGAGTTTGATCMTGGCTCAG<br>R(1492R): TACGGYTACCTTGTTACGACTT	55 °C	1500	Universal primer
16S rRNA (for sequencing)	F(785F): GGATTAGATACCCTGGTA<br>R(907R): CCGTCAATTCMTTTRAGTTT	-	-	Universal primer
sodA	F(d1): CCITAYICITAYGAYGCIYTIGARCC<br>R(d2): ARRTARTAIGCRTGYTCCCAIACRTC	37 °C	480	[1]
Antimicrobial-resistance genes				
Tetracyclines				
tet(M)	F(tetM-F): GAATCTGAACAATGGGAT<br>R(tetM-R): CTAACAATTCTGTTCCAGC	55 °C	1,099	[2]
tet(O)	F(tetO_F): AGACGGAGCAGTATTAG<br>R(tetO_R): CTGCCCAACCTTTTGCTTCAC	55 °C	200	[2]
tet(Q)	F(tetQ_F): GACTCTATGGATATAGAG<br>R(tetQ_R): CCATATCCTCTACAATCG	55 °C	835	[2]
tet(S)	F(tetS_F): CATAGACAAGCCGTTGACC<br>R(tetS_R): ATGTTTTTGGAACGCCAGAG	55 °C	667	[2]
Lincosamides				
lnu(C)	F(lnuC-F): AATTTGCAATAGATGCGGAGA<br>R(lnuC-R): TCATGTGCATTTTCATCA	55 °C	400	[3]
Macrolides				
erm(A)	F(ermA-F): ACGATATTCACGGTTTACCCACTTA<br>R(ermA-R): AACCAGAAAAACCCTAAAGACACG	53 °C	610	[4, 5]
erm(C)	F(ermC-F): TCAAAACATAATATAGATAAA	50 °C	641	[4, 5]

	R(ermC-R): GCTAATATTGTTTAAATCGTCAAT			
erm(B)	F(erm(B)F): GGTAAAGGGCATTTAACGAC<br>R(erm(B)R): CGATATTCTCGATTGACCCA	55 °C	454	[6]
mef(A)	F(mef(A)F): AGTATCATTAATCACTAGTGC<br>R(mef(A)R): TTCTTCTGGTACTAAAAGTGG	55 °C	328	[6]
Detection of the Tn916-like transposase				
Tn916-like transposase	F(Tn916L-F): GCCATGACCTATCTTATA<br>R(Tn916L-R): CTAGATTGCGTCCAA	51 °C	476	[7]
L(+)-lactate dehydrogenase gene				
ldh gene	F(ldh-F): CTTGACTCAGCTCGTTTCCG<br>R(ldh-R): GAGTAAGCAGCGTCACGAAC	55 °C	224	this study

References

1. Poyart, C.; Quesne, G.; Trieu-Cuot, P. Taxonomic dissection of the *Streptococcus bovis* group by analysis of manganese-dependent superoxide dismutase gene (*sodA*) sequences: reclassification of '*Streptococcus infantarius* subsp. *coli*' as *Streptococcus lutetiensis* sp. nov. and of *Streptococcus bovis* biotype II.2 as *Streptococcus pasteurianus* sp. nov. *Int. J. Syst. Evol. Microbiol.* **2002**, 52(4), 1247–1255. https://doi.org/10.1099/00207713-52-4-1247
2. Jun, L.J. Characterization of antibiotic resistant genes carried by fish pathogens in Korea. Doctoral dissertation, Ph. D. Thesis, Pukyong National University, Busan, Korea, 2010.
3. Lüthje, P.; Schwarz, S. Molecular basis of resistance to macrolides and lincosamides among Staphylococci and Streptococci from various animal sources collected in the resistance monitoring program BfT-GermVet. *Int. J. Antimicrob. Agents* **2007**, 29(5), 528–535.

https://doi.org/10.1016/j.ijantimicag.2006.12.016

4. Li, L.; Olsen, R.H.; Shi, L.; Ye, L.; He, J.; Meng, H. Characterization of a plasmid carrying cat, *ermB* and *tetS* genes in a foodborne *Listeria monocytogenes* strain and uptake of the plasmid by cariogenic *Streptococcus mutans*. *Int. J. Food Microbiol.* **2016**, 238, 68–71. https://doi.org/10.1016/j.ijfoodmicro.2016.08.038
5. Volokhov, D.; Chizhikov, V.; Chumakov, K.; Rasooly, A. Microarray analysis of erythromycin resistance determinants. *J. Appl. Microbiol.* **2003**, 95(4), 787-798. https://doi.org/10.1046/j.1365-2672.2003.02046.x
6. Leclercq, R.; Huet, C.; Picherot, M.; Trieu-Cuot, P.; Poyart, C. Genetic basis of antibiotic resistance in clinical isolates of *Streptococcus gallolyticus* (*Streptococcus bovis*). *Antimicrob. Agents Chemother.* **2005**, 49(4), 1646–1648. https://doi.org/10.1128/AAC.49.4.1646-1648.2005.
7. De Vries, L.E.; Christensen, H.; Skov, R.L.; Aarestrup, F.M.; Agersø, Y. (2009). Diversity of the tetracycline resistance gene *tet(M)* and identification of Tn916-and Tn5801-like (Tn6014) transposons in *Staphylococcus aureus* from humans and animals. *J. Antimicrob. Chemother.* **2009**, 64(3), 490–500. https://doi.org/10.1093/jac/dkp214

Table S3. Biochemical characterization of SBSEC used in this study

Strains	Substrate																				
	**VP**	**HIP**	**ESC**	**PYRA**	**αGAL**	**βGUR**	**βGAL**	**PAL**	**LAP**	**ADH**	**RIB**	**ARA**	**MAN**	**SOR**	**LAC**	**TRE**	**INU**	**RAF**	**AMD**	**GLYG**	**βHEM**
Streptococcus equinus																					
CNU_5	+	-	-	-	+	-	-	-	+	-	-	-	-	-	+	+	-	+	+	+	-
CNU_77-2	+	-	-	-	+	-	-	-	+	-	-	-	-	-	-	-	+	+	+	+	-
CNU_77-3	+	-	-	-	+	-	-	-	+	-	-	-	-	-	+	-	+	+	+	+	-
CNU_11	-	-	-	-	+	-	-	-	+	-	-	-	-	-	+	+	+	+	+	+	-
CNU_77-16	+	-	-	-	+	-	-	-	+	-	-	-	-	-	+	+	-	+	+	+	-
CNU_77-20	+	-	-	-	+	-	-	-	+	-	-	-	-	-	+	+	+	+	+	+	-
CNU_77-23	+	-	-	-	+	-	-	-	+	-	-	-	-	-	+	+	+	+	+	+	-
CNU_77-27	+	-	-	-	+	-	-	-	+	-	-	-	-	-	+	+	+	+	+	+	-
CNU_GF	+	-	+	-	+	-	-	-	+	-	-	-	-	-	+	+	-	+	+	+	-
CNU_G1	+	-	-	-	+	-	-	-	+	-	-	-	-	-	+	+	-	+	+	+	-
CNU_G2	+	-	+	-	+	-	-	-	+	-	-	-	-	-	+	+	-	+	+	+	-
CNU_G3	+	-	-	-	+	-	-	-	+	-	-	-	-	-	+	+	-	+	+	+	-
CNU _G4	+	-	-	-	+	-	-	-	+	-	-	-	-	-	+	+	-	+	+	+	-
CNU_G5	+	-	-	-	-	-	-	-	+	-	-	-	-	-	+	+	+	+	+	+	-
CNU_G6	+	-	+	-	+	-	-	-	+	-	-	-	-	-	+	+	-	+	+	+	-
CNU_9	+	-	-	-	+	-	-	-	+	-	-	-	-	-	+	+	-	+	+	+	-
CNU_77-8	-	-	-	-	+	-	-	-	+	-	-	-	-	-	+	+	+	+	+	+	-
CNU_77-11	+	-	-	-	+	-	-	-	+	-	-	-	-	-	+	+	-	+	+	+	-
CNU_77-12	+	-	-	-	+	-	-	-	+	-	-	-	-	-	+	+	+	+	+	+	-
CNU_77-14	-	-	-	-	+	-	-	-	+	-	-	-	-	-	+	-	-	+	+	+	-
CNU_77-29	+	-	-	-	+	-	-	-	+	-	-	-	-	-	+	+	+	+	+	+	-
CNU_15	+	-	-	-	+	-	-	-	+	-	-	-	-	-	+	+	-	+	+	+	-
CNU_20	+	-	-	-	+	-	-	-	+	-	-	-	-	-	+	+	-	+	+	+	-
CNU_21	+	-	-	-	+	-	-	-	+	-	-	-	-	-	+	+	-	+	+	+	-
CNU_77-35	+	-	-	-	+	-	-	-	+	-	-	-	-	-	+	+	-	+	+	+	-
CNU_77-37	+	-	-	-	+	-	-	-	+	-	-	-	-	-	+	+	-	+	+	+	-

CNU_77-40	+	-	+	-	+	-	-	-	+	-	-	-	-	-	+	+	+	+	+	+	-
CNU_25	+	-	-	-	+	-	-	-	+	-	-	-	-	-	+	+	-	+	+	+	-
CNU_27	+	-	-	-	-	-	+	-	+	-	-	-	-	-	+	+	-	+	+	+	-
CNU_77-43	+	-	+	-	+	-	-	-	+	-	-	-	-	-	+	+	+	+	+	+	-
CNU_77-47	+	-	-	-	+	-	-	-	+	-	-	-	-	-	+	+	-	+	+	+	-
CNU_77-50	+	-	-	-	+	-	-	-	+	-	-	-	-	-	+	+	-	+	+	+	-
CNU_29	+	-	-	-	-	-	-	-	+	-	-	-	-	-	+	-	-	+	+	+	-
CNU_30	+	-	-	-	+	-	-	-	+	-	-	-	-	-	+	-	-	+	+	+	-
CNU_32	+	-	-	-	+	-	-	-	+	-	-	-	-	-	+	+	-	+	+	+	-
CNU_77-51	+	-	-	-	+	-	-	-	+	-	-	-	-	-	+	+	-	+	+	+	-
CNU_77-55	+	-	-	-	+	-	-	-	+	-	-	-	-	-	+	-	-	+	+	+	-
CNU_77-56	+	-	-	-	+	-	-	-	+	-	-	-	-	-	+	+	-	+	+	+	-
CNU_77-57	+	-	-	-	+	-	-	-	+	-	-	-	-	-	+	+	+	+	+	+	-
CNU_77-60	+	-	-	-	+	-	-	-	+	-	-	-	-	-	+	+	-	+	+	+	-
CNU_77-68	+	-	-	-	+	-	-	-	+	-	-	-	-	-	+	+	-	+	+	+	-
CNU_41	-	-	-	-	+	-	-	-	+	-	-	-	-	-	+	-	-	+	+	+	-
CNU_42	-	-	-	-	+	-	-	-	+	-	-	-	-	-	+	-	-	+	+	+	-
CNU_77-72	+	-	-	-	+	-	-	-	+	-	-	-	-	-	+	+	+	+	+	+	-
CNU_77-77	+	-	-	-	+	-	-	-	+	-	-	-	-	-	+	+	-	+	+	+	-
CNU_77-78	+	-	-	-	+	-	-	-	+	-	-	-	-	-	+	+	-	+	+	+	-
Streptococcus lutetiensis																					
CNU_33	+	-	-	-	+	-	-	-	+	-	-	-	-	-	+	-	-	+	+	+	-
CNU_77-61	+	-	-	-	+	-	-	-	+	-	-	-	-	-	+	-	-	+	+	+	-
CNU_77-62	+	-	-	-	-	-	+	-	+	-	-	-	-	-	+	+	-	+	+	+	-
CNU_77-64	+	-	-	-	+	-	-	-	+	-	-	-	-	-	+	-	-	+	+	+	-
CNU_77-76	+	-	-	-	+	-	-	-	+	-	-	-	-	-	+	+	-	+	+	+	-

VP, Voges-Proskauer; HIP, hippuric acid; ESC, esculin; PYRA, pyrrolidonyl arylamidase; αGAL; α-galactosidase; βGUR, β-glucuronidase; βGAL, β-galactosidase; PAL, alkaline phosphatase; LAP, leucine aminopeptidase; ADH, arginine dihydrolase; RIB, ribose; ARA, arabinose; MAN, mannitol; SOR, sorbitol; LAC, lactose; TRE, trehalose; INU, inulin; RAF, raffinose; AMD, amidon; GLYG, glycogen; βHEM, β-hemolysis.
